# Supplementary material for: Carbon Ion Radiotherapy: An Evidence-Based Review and Summary Recommendations of Clinical Outcomes for Skull-Base Chordomas and Chondrosarcomas
Source: Cancers (Basel). 2023 Oct 17;15(20):5021. doi: 10.3390/cancers15205021 (PMC10605639; doi:10.3390/cancers15205021)
Supplement: Supplementary file 1 [file cancers-15-05021-s001.zip › cancers-2549188-supplementary.pdf]

Table 1. Skull-base chordoma and chondrosarcoma.

Author, Year, Institution	LOE/Study Design	No. of Pts.	Median Total Dose and/or Dose Range, GyE	Total Fxs	Median Follow-up, months (range)	Local Control	Overall Survival	Grade 3+ Toxicity or Other QOL Outcome
Chordoma								
**Lu 2020 MCR**[17]	2/Systematic review	389	48-74	16-37	NA	1, 5, and 10 yrs, 99%, 80%, and 56%	1, 5, and 10 yrs, 100%, 94%, and 78%	The incidence of early and late toxicity (Grade ≥ 3) ranged from 0 to 4% across all study groups.
**Iannalfi A 2020 CNAO**[18]	3/prospective phase 2 + prospective registry cohort	65	70.4	16	49 (6-87)	3 and 5 yrs, 77% and 71% for carbon	3 and 5 yrs, 90% and 82%	Acute: None<br>Late: gr 4 eye, 2 pts; gr 3 nervous system disorder, 3 pts. All of 3 cases of G4 toxicity were expected: in 2 patents, the optic nerve was very close to the GTV (causing visual field deficit at the baseline); in a third case, the patient was affected by preexistent unilateral important hearing deficit.
**Mizoe JE 2009 NIRS**[19]	3/prospective trial (pilot, phase 1/2, phase 2)	33	48.0 - 60.8	16	53 (8-129)	5 and 10 yrs, 85.1% and 63.8%	5 and 10 yrs, 87.7% and 67%	Acute: None<br>Late: None
**Schulz-Ertner D 2007 GSI**[20]	3/prospective phase 1/2	96	60 (60-70)	20	31 (3-91)	3 and 5 yrs, 80.6% and 70.0%	3 and 5 yrs, 91.8% and 88.5%	Acute: gr 3 mucositis, 3 pts<br>Late: gr 3 optic nerve neuropathy, 4.1%; necrosis fat plomb, 1 pt
**Mattke 2023 GSI**[21]	4/retrospective	111	66	22	49 (41-54)	3 and 5 yrs, 80% and 65% for carbon	3 and 5 yrs, 91% and 83% for carbon	Acute: None<br>Late: No grade 4
**Koto M 2020 NIRS**[22]	4/retrospective (prospectively enrolled)	34	60	16	108 (9-175)	5 and 9 yrs, 76.9% and 69.2%	5 and 9 yrs, 93.5% and 77.4%.	Acute: gr 3 mucositis, 1 pt<br>Late: gr 3 mucosal ulcer, 1 pt; gr 4 ipsilateral optic nerve injuries, 2 pts; gr 5 mucosal ulcer, 1 pt

Author, Year, Institution	LOE/Study Design	No. of Pts.	Median Total Dose and/or Dose Range, GyE	Total Fxs	Median Follow-up, months (range)	Local Control	Overall Survival	Grade 3+ Toxicity or Other QOL Outcome
**Sprave 2019 GSI[23]**	4/Cost-effectiveness	NA	NA	NA	NA	NA	NA	QALYs were 6.65 for photon RT and 8.26 for CIRT, a difference of 1.61 discounted lifetime QALYs for patients treated with CIRT. The overall ICER was €8,855.76/QALY
**Takagi M 2018 HIBMC[24]**	4/retrospective	13	57.6-74.0	16-37	56	5 and 8 yrs, 85% and 71%	5 and 8 yrs, 86% and 76%	Acute: None<br>Late: gr 3: brain necrosis, 2 pts; optic nerve disorder, 1 pt; nervous system disorders, 2 pts; middle ear inflammation, 1 pt; gr 4: pharyngeal hemorrhage, 1 pt
**Uhl M 2014 GSI[25]**	4/retrospective	155	75	20	72 (12-165)	3, 5, and 10 yrs, 82%, 72%, and 54%	3, 5, and 10 yrs, 95%, 85%, and 75%	Not graded: At 10 yrs: headache, dizziness, fatigue, xerostomia, double vision, visual deficits, hearing deficits, and cranial nerve deficits
**Jäkel 2007 GSI[26]**	4/Cost-effectiveness	10	NA	NA	NA	NA	NA	Based on a 70% or higher LC, CIRT treatment costs are lower than compared to conventional RT. The cost-effectiveness ratio for CIRT 2,539<br>Euro per 1% increase in survival, or 7,692 Euro per additional life year.
Chondrosarcoma								
**Lu 2020 MCR[17]**	2/Systematic review	243	48-80		NA	1, 5, and 10 yrs, 99%, 89%, and 88%	1, 5, and 10 yrs, 99%, 95%, and 79%	The incidence of early and late toxicity (Grade ≥ 3) ranged from 0 to 4% across all study groups.

Author, Year, Institution	LOE/Study Design	No. of Pts.	Median Total Dose and/or Dose Range, GyE	Total Fxs	Median Follow-up, months (range)	Local Control	Overall Survival	Grade 3+ Toxicity or Other QOL Outcome
**Schulz-Ertner D 2007 GSI[27]**	3/prospective phase 1/2	54	60 (57-70)	15	33 (3-84) mos	3 and 4 yrs, 96.2% and 89.8%	3 and 4 yrs, both 98.2%	Acute: gr 3 mucositis, 1 pt<br>Late: gr 3 abducent nerve paresis, 1 pt<br>4 patients experienced an improvement in preexisting cranial nerve deficits (abducent nerve paresis, 2 pts; oculomotor nerve paresis, 1 pt; facial nerve paresis, 1 pt)
**Mattke M 2018[28] and Uhl M 2014 GSI[29]**	4/retrospective	79	60 (57-69)	20	91 (3-175)	1, 2, and 4 yrs, 98.6%, 97.2%, 90.5% (Mattke)	1, 2, and 4 yrs, 100%, 98.5%, 92.9% (Mattke)	Hearing problems, 40%; cranial nerve deficit, 19%; nerve paralysis, 9%; double vision, 21% (Mattke)
Chordoma/Chondrosarcoma								
**Dong CAS 2022[30]**	2/Systematic review	781	48-80	16-25	NA	5 and 10 yrs, 74.3 and 64.7%	5 and 10 yrs, 72.7% and 72.1%	Acute: None of skull-base<br>Late: None of skull-base
**Combs SE 2009 GSI[31]**	3/prospective	17	60 (60-66.6)	20	49 (3-112)	94%	NR	Acute: None<br>Late: None<br>Not graded: ACTH and GH deficiency, 1 pt; gonadotropin deficiency, 1 pt
**Schulz-Ertner D 2002[32], 2003[33] and 2004 GSI[34]**	3/prospective phase 1/2	87	60 (60-70)	20	15 (3-46)	Ch: 3 yrs, 81%<br>Cs: 3 yrs 100%	3 yrs, 91%	None
**Guan SPHIC 2019[35]**	4/retrospective	55	63-69	21-23	28 (8-59)	2 yrs, 75.6%*	2 yrs, 87.3	Acute: 1 mucositis<br>Late: None
**Wu S 2019 SPHIC[36]**	4/retrospective	21	69 (57-80)	18-25	21.8 (7.2-39.2)	1 and 2 yrs, 93.8% and 85.2%	1 and 2 yrs, 100%	Acute: None<br>Late: None

Author, Year, Institution	LOE/Study Design	No. of Pts.	Median Total Dose and/or Dose Range, GyE	Total Fxs	Median Follow-up, months (range)	Local Control	Overall Survival	Grade 3+ Toxicity or Other QOL Outcome
**Koto M 2014 NIRS[37]**	4/retrospective	47	48.0-60.8	16	67 (24-152)	NR	NR	At 5 yrs, the likelihood of >gr 2 RT-induced brain injury and >gr 2 clinical symptoms were 24.5% and 7.0%, respectively. Brain volume receiving >50 GyE was a significant risk factor for the development of >gr 2 RT-induced brain injury.

Abbreviations: LOE, level of evidence; ACTH, adrenocorticotropic hormone; CNAO, National Center of Oncological Hadrontherapy (Italy); GH, growth hormone; gr, grade; GSI, German Ion Research Center (Heidelberg); HIBMC, Hyogo Ion Beam Medical Center (Japan); NIRS, National Institute of Radiological Sciences (Japan); MCR, Mayo Clinic Rochester; CAS, Chinese Academy of Sciences; NR, not reported; NA, not applicable; ORN, osteoradionecrosis; SNHL, sensorineural hearing loss; SPHIC, Shanghai Proton and Heavy Ion Center; yrs, years; QALY, quality-adjusted life year, ICER, incremental cost-effectiveness ratio; Cs, chondrosarcoma; Ch, chordoma; GyE, Gray equivalents; *progression-free survival.

Table 2. Re-irradiation for skull-base chordoma and chondrosarcoma.

Author, Year, Institution	LOE/Study Design	No. of Pts.	Median Total Dose and/or Dose Range, GyE	Total Fxs	Median Follow-up, months (range)	Local Control	Overall Survival	Grade 3+ Toxicity or Other QOL Outcome
Re-irradiation								
**Uhl M 2014 GSI**[38]	4/retrospective reRT	25	51 (45-60)	17	14 (2-30)	6 relapses	NR	Acute: gr 3 ORN<br>Not graded: Cranial nerve impairment remained in 6 pts and appeared in 9 pts
**Jensen 2011 GSI**[39]	4/retrospective reRT	6	44.8 (36-72.7)	NR	NA	4/5 Ch-CS stable	NA	Acute: No gr 3, only 5 (31%) acute gr 2 which resolved by first follow-up
**Combs 2011 GSI**[40]	4/retrospective reRT	18	51 (42-60)	14-20	41 (10-72)	2 and 3 yrs, 92%, and 64%	2 and 5 yrs, 86%, and 43%	Acute: Gr 1 and 2 mucositis, 5 pt; conjunctivitis, 1 pt; hearing impairment 1 pt<br>Late: None

Abbreviations: LOE, level of evidence; gr, grade; GSI, German Ion Research Center (Heidelberg); NR, not reported; NA, not applicable; yrs, years; reRT, re-irradiation.
